# Supplementary material for: Come out of Your Shell—A Comparative Pilot Study for Teaching the Central Plastrotomy in Chelonians Using a 3D-Printed Simulator and a Virtual 3D Simulation
Source: Animals (Basel). 2025 Mar 13;15(6):824. doi: 10.3390/ani15060824 (PMC11939662; doi:10.3390/ani15060824)
Supplement: Supplementary file 1 [file animals-15-00824-s001.zip › animals-3500098-supplementary.pdf]

Document S1. Translation of questionnaire - demographic data, previous knowledge, and experience (DKE)

Knowledge and Experience

Glossary:

reptiles = snakes, lizards, chelonians (turtles and tortoises)

1. Personal data:

Semester

Item 1: **In which semester are you subscribed to study veterinary medicine?**
Type of Question: single choice

- ◻ 5th semester
- ◻ 6th semester
- ◻ 7th semester
- ◻ 8th semester

Previous education/study programs

Item 2: **Did you already complete training as a veterinary assistant?**
Type of Question: single choice

- ◻ Yes
- ◻ No

Item 3: **Did you already complete a degree program related to veterinary medicine?**
Type of Question: single choice

- ◻ Yes
- ◻ No

If so, which course of study was it?
Type of Question: multiple choice/free-text response

- ◻ Biology
- ◻ Agricultural science
- ◻ Human medicine
- ◻ Medical technology
- ◻ Others:

2. Previous experience with reptiles:

Contact with reptiles at TiHo

Item 4: **Have you had any theoretical contact with reptiles during your veterinary studies (e.g., as part of lectures or clinical training on patients)?**
Type of Question: single choice

- ◻ Yes
- ◻ No

Item 5: **Have you had any hands-on contact with reptiles during your veterinary studies (e.g. ,as part of propaedeutic courses)?**
Type of Question: single choice

- ◻ Yes
- ◻ No

Contact with reptiles in a private setting

Item 6: **Do you have any contact with reptiles in your private life (e.g., as pets)?**
Type of Question: single choice

- ◻ Yes
- ◻ No

If you have contact with reptiles in your private environment, please name the type of reptile(s) involved
Type of Question: multiple choice

- ◻ Snake
- ◻ Lizard
- ◻ Chelonian

3. Previous experience of surgical interventions on reptiles:

General experience of surgical interventions

Item 7: **During which of the following surgical interventions on a reptile were you already present?**
Type of Question: multiple choice/free-text response

- □ Castration (removal of the reproductive organs)
- □ Amputation (removal of body parts)
- □ Wound management
- □ Tumor extraction (removal of circumferential growth)
- □ Prolapse reduction (repositioning of prolapsed organs)
- □ Fracture treatment (treatment of bone fractures)
- □ Others:

Item 8: **Which of the surgical interventions on a reptile listed below did you already perform yourself?**
Type of Question: multiple choice

- □ Castration (removal of the reproductive organs)
- □ Amputation (removal of body parts)
- □ Wound management
- □ Tumor extraction (removal of circumferential growth)
- □ Prolapse reduction (repositioning of prolapsed organs)
- □ Fracture treatment (treatment of bone fractures)
- □ Others:

Coeliotomy

Item 9: **What do you understand by the term coeliotomy in reptiles?**
Type of Question: single choice

- □ Incision of the coelomial cavity in reptiles
- □ Incision of the plastron in reptiles
- □ Incision of organs in reptiles
- □ Incision of the coelomial cavity and incision of organs in reptiles
- □ Incision of the plastron and incision of the coelomial cavity in reptiles

Item 10: **Did you already perform a coeliotomy on reptiles under the guidance of a veterinarian?**
Type of Question: single choice

- □ Yes
- □ No

Item 11: **Did you already perform a coeliotomy on reptiles <u>independently without guidance</u> from a veterinarian?**
Type of Question: single choice

◻ Yes<br>◻ No

<u>Plastrotomy</u>

Item 12: **What do you understand by the term plastrotomy in reptiles?**
Type of Question: single choice

◻ Incision of the coelomial cavity in reptiles<br>◻ Incision of the plastron in reptiles<br>◻ Incision of organs in reptiles<br>◻ Incision of the coelomial cavity and incision of organs in reptiles<br>◻ Incision of the plastron and incision of the coelomial cavity in reptiles

Item 13: **For which of these indications is a plastrotomy indicated?**
Type of Question: multiple choice

◻ Dystocia (difficult/impeded egg laying in reptiles & birds)<br>◻ Cystic calculi<br>◻ Limb amputation<br>◻ Foreign bodies in the gastrointestinal tract<br>◻ Prolapse<br>◻ Castration

Item 14: **Did you already perform a plastrotomy on reptiles independently without guidance from a veterinarian?**
Type of Question: single choice

◻ Yes<br>◻ No

Item 15: **Did you already perform a coeliotomy on reptiles independently without guidance from a veterinarian?**
Type of Question: single choice

◻ Yes<br>◻ No

4. Participation in electives

Item 16: **Did you already take part in the Reptile Surgery Elective at the Department of Small Mammal, Reptile and Avian Medicine and Surgery at the University of Veterinary Medicine Hannover, Foundation?**
Type of Question: single choice

□ Yes<br>□ No

5. Participation in exercises in the Clinical Skills Lab

Item 17: **Have you ever attended exercises in the Clinical Skills Lab at the University of Veterinary Medicine Hannover, Foundation?**
Type of Question: single choice

□ Yes<br>□ No

If you attended exercises in the Clinical Skills Lab at the University of Veterinary Medicine Hannover, Foundation, please tick the appropriate type of course:
Type of Question: multiple choice

□ Courses with focus on surgery<br>□ Courses with focus on laboratory area<br>□ Courses with focus on cattle<br>□ Courses with focus on horses<br>□ Courses with focus on small cloven-hoofed animals<br>□ Courses with focus on small animals<br>□ Courses with focus on pets<br>□ Courses with focus on birds<br>□ Courses with focus on reptiles

Item 18: **Did you prepare for the exercises in the Clinical Skills Lab at home?**
Type of Question: single choice

□ Yes<br>□ No

If you attended exercises in the Clinical Skills Lab at the University of Veterinary Medicine Hannover, Foundation, please tick the appropriate type of study material you used:

Type of Question: multiple choice/free-text response

- □ Textbooks
- □ YouTube videos (Clinical Skills Lab channel of the University of Veterinary Medicine Hannover, Foundation)
- □ Learning material from lectures
- □ Professional journals
- □ Student committee script (AstA)
- □ Internet material
- □ Other:

Thank you for taking the time to complete this survey.

Document S2. Translation of questionnaire on self-efficacy (self-assessment of skills) (SE)

Self-efficacy

Glossary:

reptiles = snakes, lizards, chelonians (turtles and tortoises)

coelomic cavity = reptiles have one contiguous body cavity. Unlike mammals, this body cavity is not divided into a thoracic and an abdominal cavity by a diaphragm. The organs are all located in one body cavity, the so-called coelomic cavity.

coeliotomy = opening the coelomic cavity by incision

plastrotomy = surgical procedure in reptiles that involves opening the plastron (abdominal shell) of the chelonian by cutting through the horn plates and opening the coelomic cavity by incision.

Type of Question: single choice

		I strongly agree	I agree	I disagree	I strongly disagree	No indication
1	I feel well prepared in theory to perform a plastrotomy on a chelonian					
2	I know the steps involved in preparing, performing, and post-processing a plastrotomy on a chelonian					

3	I can visualize these steps well in my mind					
4	I can determine a medical indication (e.g., dystocia, foreign bodies in the gastrointestinal tract, cystic calculi) for performing a plastrotomy on a chelonian based on a radiographic examination					
5	I am able to select the surgical field for a plastrotomy in a chelonian based on radiographs and to mark it on the plastron					
6	I know which surgical instruments and materials I will need to be prepared to perform a plastrotomy on a chelonian					
7	I am able to correctly estimate the **penetration depth** of the cutting disk to correctly perform a plastrotomy on a chelonian					
8	I am able to correctly estimate the **angle** of the cutting disk to correctly perform a plastrotomy on a chelonian					
9	I am able to correctly name anatomically relevant structures within the coelomic cavity of a chelonian					
10	I know which organs and structures can be injured if a chelonian's plastrotomy is not performed correctly					

		I strongly agree	I agree	I disagree	I strongly disagree	No indication
11	I have the confidence to professionally close the abdominal shell of a chelonian after performing a plastrotomy					
12	I have the confidence to perform a plastrotomy on chelonian **cadaver** myself under professional veterinary supervision and guidance					
13	I have the confidence to perform a plastrotomy on a chelonian **cadaver** myself **without** professional veterinary supervision and guidance					
14	I have the confidence to perform a plastrotomy on **living** chelonian myself under professional veterinary supervision and guidance					

15	I have the confidence to perform a plastrotomy on **living** chelonian myself **without** professional veterinary supervision and guidance					

Thank you for taking the time to complete this survey.

Document S3. Translation of questionnaire on evaluation for students (ES1)

Evaluation of Simulator/Simulation

Glossary:

reptiles = snakes, lizards, chelonians (turtles and tortoises)

plastrotomy = surgical procedure in reptiles that involves opening the plastron (abdominal shell) of the chelonian by cutting through the horn plates and opening the coelomic cavity by incision.

Item 1: **Which simulator/simulation did you use in the training course?**
Type of Question: single choice

- ◻ 3D-printed simulator
- ◻ Virtual 3D simulation

Item 2: **How do you generally enjoy learning how to perform a plastrotomy in chelonians using a simulator/simulation?**
Type of Question: single choice

- ◻ Good
- ◻ Mediocre
- ◻ Poor
- ◻ Not applicable

Item 3: **Do you think it is useful to train the procedure of a plastrotomy on a simulator/simulation before performing a plastrotomy on a chelonian <u>cadaver</u>?**
Type of Question: single choice

- ◻ Yes
- ◻ No
- ◻ Not applicable

Item 4: **Do you think it is useful to train the procedure of a plastrotomy on a simulator/simulation before performing a plastrotomy on a <u>live</u> chelonian?**
Type of Question: single choice

- ◻ Yes
- ◻ No
- ◻ Not applicable

Item 5: **Which of the available resources do you want to use to prepare for a plastrotomy in chelonians?**

Type of Question: single choice

- ◻ 3D-printed simulator
- ◻ Virtual 3D simulation
- ◻ Combination of 3D-printed simulator and virtual 3D simulation
- ◻ Not applicable

Item 6: **Do you want more simulators/simulations concerning these subjects (reptiles, surgery) to be implemented in the study of veterinary medicine in the future?**

Type of Question: single choice

- ◻ Yes
- ◻ No
- ◻ Not applicable

Item 7: **What do you like on the used simulators/simulations?**

Type of Question: Free-text response

Item 8: **How would you improve the used simulators/simulations?**

Type of Question: Free-text response

Item 9: **Do you have any further comments or suggestions?**
Type of Question: Free-text response

Please comment on the following statements:
Type of Question: single choice

**Suitability and areas of application of the used resource**						
		I strongly agree	I agree	I disagree	I strongly disagree	No indication
1	This simulator/simulation realistically depicts the conditions during a plastrotomy in chelonians					
2	This simulator/simulation offers a good overview of the anatomy of chelonians					
3	This simulator/simulation is unrealistic					
4	This simulator/simulation can be used to perform hands-on training of a plastrotomy in chelonians in the study of veterinary medicine (for **students**)					
5	This simulator/simulation can be used to perform hands-on training of a plastrotomy in chelonians as part of veterinary training/continuing education (for **veterinarians**)					
6	This simulator/simulation can be used to provide hands-on training for other surgical procedures on chelonians (e.g., removal of cystic calculi or foreign bodies in the gastrointestinal tract)					

**Effects of the used resource**						
		I strongly agree	I agree	I disagree	I strongly disagree	No indication
1	This simulator/simulation improves the **learning success** of the trainees when performing a plastrotomy in chelonians					
2	This simulator/simulation improves the **self-confidence** of the trainees when performing a plastrotomy in chelonians					
3	This simulator/simulation improves the **safety** of the trainees when performing a plastrotomy in chelonians					

**Comparison with other teaching methods**						
		I strongly agree	I agree	I disagree	I strongly disagree	No indication
1	This simulator/simulation is a **useful addition** to a hands-on training on cadavers in preparation for a plastrotomy in chelonians					
2	This simulator/simulation is a **useful substitute** for a hands-on training on cadavers in preparation for a plastrotomy in chelonians					
3	This simulator/simulation is **not a useful extension** of existing resources in preparation for a plastrotomy in chelonians					
4	This simulator/simulation can be combined well with other resources in preparation for a plastrotomy in chelonians					

Please rate the following criteria regarding how realistic the simulator/simulation is:
Type of Question: single choice

		Very good	Good	Satisfactory	Sufficient	Poor	No indication
1	Quality						
2	Functionality						
3	Reality						
4	User-friendliness						
5	Reusability						
6	(Appropriate) level of difficulty						
7	Anatomical correctness						
8	Visibility of landmarks						
9	Optics						
10	Size						
11	Haptic						

Thank you for taking the time to complete this survey.

Document S4. Translation of questionnaire on evaluation for clinicians (ES2)

Evaluation

Glossary:

reptiles = snakes, lizards, chelonians (turtles and tortoises)

plastrotomy = surgical procedure in reptiles that involves opening the plastron (abdominal shell) of the chelonian by cutting through the horn plates and opening the coelomic cavity by incision

Item 1: **Which of the following (job) titles applies to you?**
Type of Question: multiple choice

◻ Veterinary student<br>◻ Teaching staff<br>◻ Practitioner<br>◻ Other:

Item 2: **Which simulator/simulation did you use in the training course?**
Type of Question: single choice

◻ 3D-printed simulator<br>◻ Virtual 3D simulation

Item 3: **How do you generally enjoy learning how to perform a plastrotomy on chelonians using a simulator/simulation?**
Type of Question: single choice

◻ Good<br>◻ Mediocre<br>◻ Poor<br>◻ Not applicable

Item 4: **Do you think it is useful to train the procedure of a plastrotomy using a simulator/simulation before performing a plastrotomy on a chelonian <u>cadaver</u>?**
Type of Question: single choice

◻ Yes<br>◻ No<br>◻ Not applicable

Item 5: **Do you think it is useful to train the procedure of a plastrotomy using a simulator/simulation before performing a plastrotomy on a <u>live</u> chelonian?**
Type of Question: single choice

- ◻ Yes
- ◻ No
- ◻ Not applicable

Item 6: **Which of the available resources do you want to use to prepare for a plastrotomy on chelonians?**

Type of Question: single choice

- ◻ 3D-printed simulator
- ◻ Virtual 3D simulation
- ◻ Combination of 3D-printed simulator and virtual 3D simulation
- ◻ Not applicable

Item 7: **Do you want more simulators/simulations concerning these subjects (reptiles, surgery) to be implemented in the study of veterinary medicine in the future?**

Type of Question: single choice

- ◻ Yes
- ◻ No
- ◻ Not applicable

Item 8: **What do you like on the used simulators/simulations?**

Type of Question: free-text response

Item 9: **How would you improve the used simulators/simulations?**

Type of Question: free-text response

Item 10: **Do you have any further comments or suggestions?**

Type of Question: free-text response

Please comment on the following statements:

Type of Question: single choice

**Suitability and areas of application of the used resource**						
		I strongly agree	I agree	I disagree	I strongly disagree	No indication
1	This simulator/simulation realistically depicts the conditions during a plastrotomy on chelonians					
2	This simulator/simulation offers a good overview of the anatomy of chelonians					
3	This simulator/simulation is unrealistic					
4	This simulator/simulation can be used to perform hands-on training of a plastrotomy on chelonians in the study of veterinary medicine (for **students**)					
5	This simulator/simulation can be used to perform hands-on training of a plastrotomy on chelonians as part of veterinary training/continuing education (for **veterinarians**)					
6	This simulator/simulation can be used to provide hands-on training for other surgical procedures on chelonians (e.g., removal of cystic calculi or foreign bodies in the gastrointestinal tract)					

Effects of the used resource						
		I strongly agree	I agree	I disagree	I strongly disagree	No indication
1	This simulator/simulation improves the **learning success** of the trainees when performing a plastrotomy on chelonians					
2	This simulator/simulation improves the **self-confidence** of the trainees when performing a plastrotomy on chelonians					
3	This simulator/simulation improves the **safety** of the trainees when performing a plastrotomy on chelonians					

Comparison with other teaching methods						
		I strongly agree	I agree	I disagree	I strongly disagree	No indication
1	This simulator/simulation is a **useful addition** to a hands-on training on cadavers in preparation for a plastrotomy on chelonians					
2	This simulator/simulation is a **useful substitute** for a hands-on training on cadavers in preparation for a plastrotomy on chelonians					
3	This simulator/simulation is **not a useful extension** of existing resources in preparation for a plastrotomy in chelonians					
4	This simulator/simulation can be combined well with other resources in preparation for a plastrotomy on chelonians					

Please rate the following criteria regarding how realistic the simulator/simulation is
Type of Question: single choice

		Very good	Good	Satisfactory	Sufficient	Poor	No indication
1	Quality						
2	Functionality						
3	Reality						
4	User-friendliness						
5	Reusability						
6	(Appropriate) level of difficulty						
7	Anatomical correctness						
8	Visibility of landmarks						
9	Optics						
10	Size						
11	Haptic						

Thank you for taking the time to complete this survey.

Document S5. Translation of the Objective Structured Clinical Examination (OSCE) checklist (CL)

Checklist

Glossary:

Coelomic cavity = reptiles have one contiguous body cavity. Unlike mammals, this body cavity is not divided into a thoracic and an abdominal cavity by a diaphragm. The organs are all located in one body cavity, the so-called coelomic cavity.

Plastron = dorsal shell of a chelonian

Carapace = ventral shell of a chelonian

Plastrotomy = surgical procedure in reptiles that involves opening the plastron (abdominal shell) of the chelonian by cutting through the horn plates and opening the coelomic cavity by incision.

Bone flap = a term used in surgery in which an area of bone is incised on three sides so that it is still attached to the fourth side; the piece of bone can be folded over at this point. This technique promises better healing, as the tissue is permanently supplied with blood via the fourth, preserved side of the bone.

	**What the examinee should do**	**Not fulfilled**	**Partially fulfilled**	**Ful-filled**	**Score**
	**Preparation for plastrotomy (preoperative)**				
1.	Prepare all necessary materials for the **preparation** of the plastrotomy <u>before</u> carrying out the task *(gloves, waterproof pen, safety glasses)*				**1**
2.	Select the correct radiographic image for indication of plastrotomy *(image A, which shows a dystocia and thus an indication of a plastrotomy)*				**2**
3.	**Mention** that the chelonian is sedated **and** intubated				**1**
4.	Position the chelonian correctly for a plastrotomy 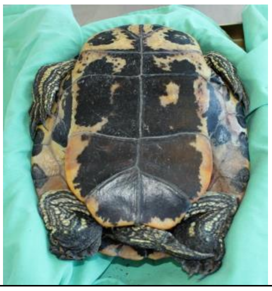				**2**

	(chelonian is placed on carapace in dorsal recumbency on surgical drape with heat pads placed on the side, the animal's head facing away from the surgeon)				
5.	Mark the surgical field on the plastron in the correct position<br>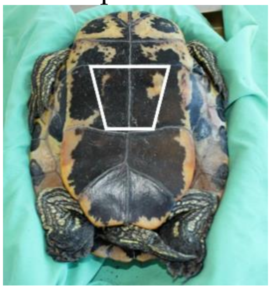<br>*(trapezoidal starting at the pectoral shield (pectorale) to just below the abdominal shield (abdominale); oriented to bone sutures (suturae); size of cut-out adapted to body size of chelonian)*				**3**
6.	**Mention or demonstrate** the cleaning of the surgical field				**1**
7.	Put on safety glasses				**1**
8.	Put on gloves (non-sterile)				**1**
	**What the examinee should do**	**Not fulfilled**	**Partly fulfilled**	**Fulfilled**	**Score**
	**Conducting the plastrotomy (intraoperative)**				
9.	Prepare all necessary materials for the **conduction** of the plastrotomy in a kidney dish <u>before</u> carrying out the task<br>*(hammer, chisel, dremel including cutting disk, gauze swabs, anatomical forceps, scissors (blunt, blunt, straight))*				**1**
10.	Position the cutting disk at the correct position<br>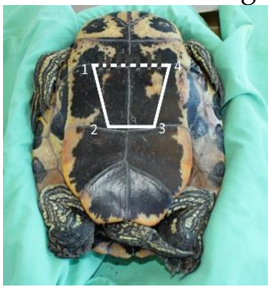<br>*(starting point: position 1)*				**2**
11.	Position the cutting disk at the correct angle<br>*(caudal and cranial: 70-80°, lateral: 60°)*				**4**
12.	**Mention that precautions are taken** to ensure that the cutting disk is cooled with sterile saline solution by an assistant				**1**

13.	Make **four** incisions in the plastron<br>*(device switched on at the highest frequency, sawing in a line if possible, three complete incisions and one incomplete incision on the cranial side to create a bone flap on the remaining side = dashed line)*				**4**
14.	Remove the horn shavings with gauze swabs				**1**
15.	Loosen the cut-out at the pre-perforated incisions using hammer and chisel<br>*(Placing the chisel under the pre-perforated incision with the non-dominant hand, hold the hammer in the dominant hand and carefully tap the chisel with the hammer until the cut-out can be lifted at the pre-perforated incisions)*				**4**
16.	Open up the bone flap cranially				**2**
17.	**Mention that precautions are taken** to ensure that the bone flap is kept permanently moist by an assistant during surgery				**1**
18.	Show access to the coelomic cavity using scissors and forceps under visual control				**2**
	**What the examinee should do**	**Not fulfilled**	**Partly fulfilled**	**Fulfilled**	**Score**
19.	Hold the forceps always in the pen grip				**1**
20.	Hold the scissors always in the ring finger-thumb grip				**1**
21.	Ensure that the coelomic cavity was opened while sparing the organs<br>*(no perforation!)*				**3**
22.	**Mention** that the coelomic cavity has to be sutured				**1**
23.	Fold the bone flap caudally				**2**
24.	The chelonian was not moved during surgery				**3**

	**Post-processing of plastrotomy (postoperative)**				
25.	Prepare all necessary materials in a kidney dish for the **post-processing** of the plastrotomy <u>before</u> performing the task<br>*(bone wax, acetone, gauze swabs, fiberglass mesh, craft scissors, paper cup, polyester resin, hardener, wooden spatula)*				**1**
26.	Complete closure of all **four** incisions by inserting bone wax				**3**
27.	Clean the plastron with a gauze swap soaked in acetone				**1**
28.	Cut the fiberglass fabric to the right size<br>*(plastron cut-out must be completely covered)*				**2**
29.	Place the cut piece of fiberglass fabric on the cut-out on the plastron				**1**
30.	Mix polyester resin and hardener in the cup using a wooden spatula in the correct proportion<br>*(polyester resin: "walnut size" to hardener: "pea size")*				**3**
31.	Apply and spread the polyester resin and hardener mixture onto the plastron and glass fiber fabric at the right moment<br>*(approx. 15 seconds before curing, when the mixture takes on a viscous consistency)*<br><br>**What the examinee should do**				**4**
		**Not fulfilled**	**Partly fulfilled**	**Fulfilled**	**Score**
	**General**				
32.	Perform the clinical skills in the correct order				**3**
	**Total**				**63**

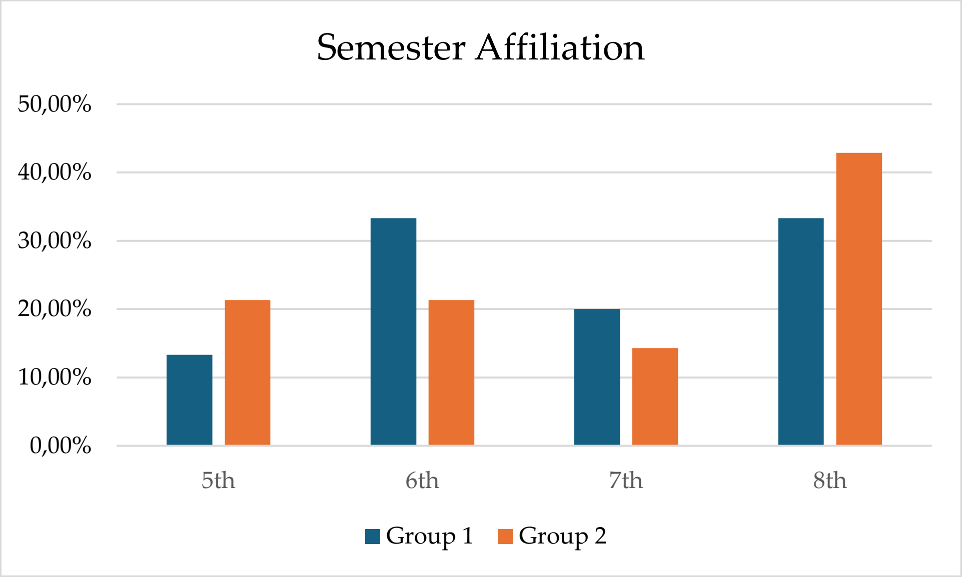

Figure S1. Semester affiliation by (group 1:3D-printed simulator, Group 2: virtual 3D simulation)

**Group**	**n**	**SE Mean Value**	**SE Standard Deviation**	**SE Minimum Points**	**SE Maximum Points**	**SE Median Points**
1	15	3.60	0.41	2	4	4
2	14	**3.57**	0.73	3	4	4

Figure S2. Self-assessment of skills (SE) of item 6 (confidence in the preparation of surgical instruments and materials) before the preparation course (t1) for both student groups (group 1: 3D-printed simulator, group 2: virtual 3D simulation).

**Group**	**n**	**SE Mean Value**	**SE Standard Deviation**	**SE Minimum Points**	**SE Maximum Points**	**SE Median Points**
1	15	**1.80**	0.41	1	2	2
2	14	2.28	0.73	1	3	2

Figure S3. Self-assessment of skills (SE) of item 7 (confidence in correct estimation of the depth of the cutting disk) after the preparation course and prior to the objective structured clinical examination (t2) for both student groups (group 1: 3D-printed simulator, group 2: virtual 3D simulation).

**Group**	**n**	**SE Mean Value**	**SE Standard Deviation**	**SE Minimum points**	**SE Maximum points**	**SE Median points**
1	15	**1.33**	0.41	1	2	2
2	13	1.07	0.73	1	2	1

Figure S4. Self-assessment of skills (SE) of item 4 confidence in the determination of a medical indication for performing a plastrotomy based on a radiographic examination after (t3) the objective structured clinical examination (OSCE) for both student groups (group 1: 3D-printed simulator, group 2: virtual 3D simulation).
